# Clinical Significance of Varying Degrees of Vancomycin Susceptilibity in Methicillin-Resistant *Staphylococcus aureus* Bacteremia^1^

**DOI:** 10.3201/eid0906.030001

**Published:** 2003-06

**Authors:** Mitchell J. Schwaber, Sharon B. Wright, Yehuda Carmeli, Lata Venkataraman, Paola C. DeGirolami, Aneta Gramatikova, Trish M. Perl, George Sakoulas, Howard S. Gold

**Affiliations:** * Beth Israel Deaconess Medical Center, Boston, Massachusetts, USA; † Harvard Medical School, Boston, Massachusetts, USA; ‡ Tel Aviv Sourasky Medical Center, Tel Aviv, Israel; § Johns Hopkins Hospital, Johns Hopkins Medical Institutions, Baltimore, Maryland, USA

**Keywords:** *Staphylococcus aureus*, methicillin resistance, vancomycin resistance, drug resistance, bacterial, bacteremia, bacterial infections, research

## Abstract

We conducted a retrospective study of the clinical aspects of bacteremia caused by methicillin-resistant *Staphylococcus aureus* (MRSA) with heterogeneously reduced susceptibility to vancomycin. Bloodstream MRSA isolates were screened for reduced susceptibility by using brain-heart infusion agar, including 4 mg/L vancomycin with and without 4% NaCl. Patients whose isolates exhibited growth (case-patients) were compared with those whose isolates did not (controls) for demographics, coexisting chronic conditions, hospital events, antibiotic exposures, and outcomes. Sixty-one (41%) of 149 isolates exhibited growth. Subclones from 46 (75%) of these had a higher MIC of vancomycin than did their parent isolates. No isolates met criteria for vancomycin heteroresistance. No differences in potential predictors or in outcomes were found between case-patients and controls. These data show that patients with vancomycin-susceptible MRSA bacteremia have similar baseline clinical features and outcomes whether or not their bacterial isolates exhibit growth on screening media containing vancomycin.

*Staphylococcus aureus* is an important cause of illness and death and accounts for about one-fifth of bacteremia cases in the United States (*1*). In 1997, Hiramatsu et al. reported the first clinical strain of methicillin-resistant *S. aureus* (MRSA) that exhibited reduced susceptibility to vancomycin (*2*). A report of other such isolates, classified as vancomycin-intermediate *S. aureus* (VISA), soon followed (*1*). Infection with VISA has been associated with vancomycin treatment failures, but it is a rare phenomenon, with worldwide prevalence limited to isolated case reports and a single limited outbreak (*1*,*3*). Rarer still in *S. aureus* is the phenomenon of vancomycin resistance (VRSA), with only two clinical VRSA isolates reported to date, both in 2002 (*4*,*5*).

Far more common than VRSA and VISA, however, are MRSA isolates that exhibit “heteroresistance” to vancomycin (hetero-VISA), whereby subpopulations within the strain exhibit reduced susceptibility, although the overall MIC for the isolate is within the susceptible range (<4 mg/L). A recent survey in Japan found this phenotype in up to 26% of clinical MRSA isolates collected in university hospitals (*6*).

Clinical laboratories do not perform heteroresistance testing for a number of reasons: Such testing is cumbersome, no standardized testing methods exist, and, perhaps most important, the clinical significance of this phenotype is not known (*7*). Although clinical MRSA isolates exhibiting hetero-VISA have now been reported from several countries (*7*–*16*), no study has demonstrated that patients with infections caused by these strains fare differently than patients with comparable infections caused by MRSA strains that are homogeneously susceptible to vancomycin.

Throughout this article, we use the following terms to describe different phenotypic features of *S. aureus* isolates: Vancomycin-susceptible *S. aureus* (VSSA) refers to isolates that are susceptible to vancomycin, according to NCCLS) criteria (MIC <4 mg/L) (*1*). VISA refers to isolates that have intermediate susceptibility to vancomycin per NCCLS criteria (MIC 8–16 mg/L) (*17*). Hetero-VISA refers to isolates for which the MIC of vancomycin for one or more subpopulations is >4 mg/L, whereas the overall MIC is <4 mg/L. VRSA refers to isolates with an MIC of vancomycin >32 mg/L (*17*).

Many investigators who have looked at hetero-VISA (so named because the resistant subclones have intermediate susceptibility) have first screened for reduced susceptibility to vancomycin and then confirmed hetero-VISA status by demonstrating MICs above the susceptible range (i.e., >4 mg/L) among subclones of those isolates with positive screening results (*6*,*9*,*10*,*12*–*16*). No study has examined isolates that meet screening criteria yet fail to qualify as hetero-VISA on confirmatory testing. Such isolates are composed of subpopulations that, although susceptible to vancomycin, demonstrate varying degrees of susceptibility. Thus they may be capable of a certain degree of growth on screening media containing vancomycin, despite an absence of subpopulations that demonstrate intermediate resistance to vancomycin by MIC criteria. If, as has been suggested, VISA arise from homogeneously vancomycin-susceptible *S. aureus* through a multistep process (*18*), hetero-VISA and, ultimately, VISA may be selected from just such a population of isolates that display heterogeneously reduced susceptibility to vancomycin but do not meet criteria for hetero-VISA. We studied patients with MRSA bacteremia in our institutions to determine the prevalence, among the infecting strains, of hetero-VISA, and of nonhetero-VISA isolates that nevertheless exhibited varying degrees of susceptibility to vancomycin among subpopulations. Additional objectives were to identify factors predictive of bacteremia with such isolates and to determine whether bacteremia with such isolates affected patient outcomes.

## Methods

### Microbiologic Methods

#### Bacterial Isolates

MRSA bloodstream isolates that had been stored nonselectively at Beth Israel Deaconess Medical Center from September 1998 through November 2001 and nosocomial bloodstream isolates from patients in intensive care units at Johns Hopkins Hospital from July 1997 through April 2000 were used. In addition, the following strains were used as controls: ATCC 29213 (MRSA), ATCC 33591 (vancomycin-susceptible MRSA), ATCC 51299 (vancomycin-resistant *Enterococcus faecium*) and PC3 (VISA strain contributed by A. Tomasz) (*19*).

### Screening for Heterogeneously Reduced Susceptibility to Vancomycin

Suspensions of 0.5 McFarland turbidity standard in brain heart infusion (BHI) broth were prepared from isolates after overnight incubation. Ten microliters of each suspension was injected onto BHI agar plates containing 4 mg/L vancomycin. Because of the reported inducibility of vancomycin heteroresistance in some strains of *S. aureus* by NaCl (*13*), each isolate was screened on agar with and without 4% NaCl supplementation.

Plates were incubated at 35°C. Results were recorded after 24 h and 48 h of incubation. For any growth in excess of a single pinpoint colony on screening media, either with or without 4% NaCl supplementation, a positive result was recorded.

### Susceptibility Testing

Susceptibility to vancomycin was determined by agar dilution, according to NCCLS guidelines (*17*), with Mueller-Hinton agar. Testing was performed on all MRSA bloodstream isolates (parent isolates), as well as on those colonies that grew on screening media (subclones). MIC testing was performed on colonies taken from the screening agar on which they exhibited optimal growth (i.e., BHI agar containing 4 mg/L vancomycin, with or without 4% NaCl).

### Identity Confirmation and Strain Typing

The identity of parent isolates and subclones was confirmed by Gram stain, catalase testing (*20*), and latex agglutination testing (Staphaurex, Murex Biotech, Ltd., Dartford, UK). Strains were grouped by type and subtype by using pulsed-field gel electrophoresis (PFGE) (*21*). Plugs were made by using standard techniques. Macrorestriction was performed with *Sma*I (*22*).

### Population Analysis

Suspensions of 3.0 McFarland turbidity standard in BHI broth were made from overnight cultures of selected parent isolates and subclones. Subclones were grown in 4 mg/L vancomycin before suspension in broth. Seven serial 10-fold dilutions of each suspension were prepared. Twenty-five microliters of each suspension and dilution was injected twice onto BHI agar containing 4% NaCl, at vancomycin concentrations of 0 mg/L, 1 mg/L, 2 mg/L, 4 mg/L, and 8 mg/L. NaCl was added due to the enhanced growth we observed on NaCl-containing media during screening. Colonies were counted after 48 h of incubation at 35°C, and sums of each inoculum pair were averaged.

### Epidemiologic Methods

#### Clinical Data Collection and Inclusion Criteria

For the epidemiologic analysis, the unit of observation shifted from the bacterial isolate to the patient with bacteremia. Demographic and clinical data for the patients with saved MRSA bloodstream isolates were collected from electronic medical records and from hospital databases. A patient could be included in the cohort only once, regardless of the number of isolates generated during the study period. Patients with multiple isolates were included at the time MRSA was first isolated from the bloodstream and the isolate grew on screening media. Those patients whose isolates never grew on screening media were included at the time of their first MRSA bloodstream isolation. Inpatients only were considered in the analysis. The study was approved by the ethics review boards of Beth Israel Deaconess Medical Center and of Johns Hopkins Hospital and performed at both institutions.

### Study Design

We conducted a two-part retrospective analysis on patients with MRSA bacteremia: a case-control analysis, which considered the clinical features at the time of culture, and a cohort analysis, which evaluated outcomes after culturing.

For the case-control study, patients whose MRSA bloodstream isolates exhibited growth on screening media (case-patients) were compared with patients whose isolates exhibited no growth (controls), in terms of the following features: age, sex, coexisting chronic conditions, hospital events, and antibiotic exposures before culture. Particular attention was given to exposure to vancomycin, examined both as a dichotomous variable and for cumulative days of exposure.

In the cohort study, we compared the above two groups of participants for the following postculture outcomes: deaths, discharge disposition, and duration of hospital stay after culture. Possible confounding variables were evaluated through multivariate modeling.

### Statistical Analysis

Statistical analyses were performed by using SAS statistical software (version 8e, SAS Institute, Inc., Cary, NC). Continuous variables were compared by using the Student t test or the Wilcoxon rank sum test, depending on the normality of the distribution. Binary variables were compared by using the Fisher exact test. Nonbinary categorical variables were compared by using the chi-square test. Multivariate logistic regression models were used in the death and disposition analyses to control for confounding. A Cox proportional hazards model was used in the time-to-discharge analysis; observations were censored at patient’s death. For all statistical tests, a p value of <0.05 was considered significant.

## Results

### Microbiologic Results

We tested 173 MRSA bloodstream isolates from 154 patients. For the following reasons, we excluded 24 isolates from the analysis: 19 represented additional isolates from patients already included in the cohort, 4 came from outpatients, and 1 came from a patient whose hospital stay extended beyond the study period. Thus, we evaluated 149 isolates, each cultured from the blood of a unique inpatient, and will describe them here.

All isolates were susceptible to vancomycin (MIC_50_, 1 mg/L; MIC_90_, 1 mg/L; range 0.5–2 mg/L). Isolates from 61 patients (41% of the patient cohort) grew on screening media within 48 h. Subclones of 46 of these isolates (75%) exhibited a two- to fourfold increase in MIC compared with the parent strain. No subclones, however, exceeded the 4 mg/L NCCLS breakpoint for vancomycin susceptibility (the MIC of vancomycin for all but one subclone was <2 mg/L; for one subclone it was 4 mg/L) (*1*). We therefore did not characterize any of the isolates as hetero-VISA. We characterized isolates with positive results on our screening assay as having heterogeneously reduced susceptibility to vancomycin.

**Figure 1:**
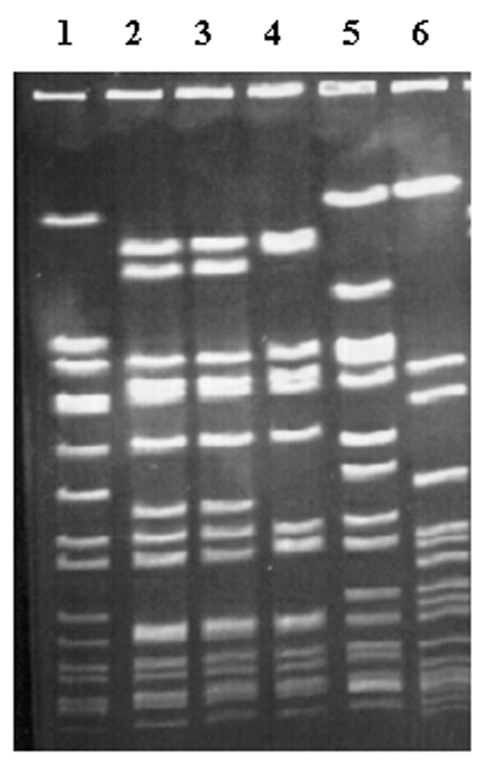
Pulsed-field gel electrophoresis of selected isolates, demonstrating predominant and secondary types. Lane 1, *Staphylococcus aureus* NCTC 8325, used as DNA molecular weight reference marker; lanes 2 and 3, clinical isolates of type A13; lane 4, clinical isolate of type A1; lane 5, clinical isolate of type B2; lane 6, clinical isolate of type H.

The isolates comprised 11 different PFGE types. We assigned letters and numbers to PFGE types and subtypes, respectively. One hundred and three (69%) were variants of type A, which had 35 different subtypes. Twenty-two (15%) were variants of type B, which had seven different subtypes. The remaining 24 isolates (16%) consisted of nine different types. Type A was associated with the absence of heterogeneously reduced susceptibility (odds ratio [OR] 0.35; p=0.004), whereas type B was associated with its presence (OR 4.86; p=0.002). The results of strain typing and the relationship between type and screening results are summarized in Table 1. Figure 1 shows the results of PFGE performed on several isolates.

**Table 1:** Strain typing and relationship to reduced vancomycin susceptibility screening results

	No. isolates	Positive results on screening	Odds ratio (95% confidence interval)	p
Type A	103	34 (33%)	0.35 (0.17 to 0.71)	0.004
Type B	22	16 (73%)	4.86 (1.87 to 13.29)	0.002
Other type	24	11 (46)	-	-

**Figure 2:**
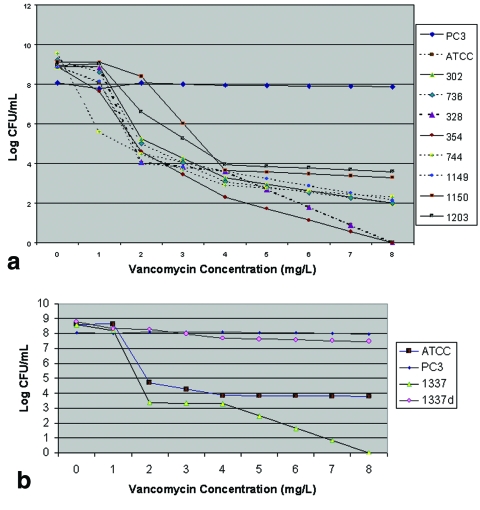
a) Population analysis of parent isolates and reference strains. PC3, vancomycin- intermediate *Staphylococcus aureus* (VISA) reference strain (published vancomycin MIC 8 mg/L (*19*); MIC 4 mg/L by our assay); ATCC, methicillin-resistant *S. aureus* (MRSA) reference strain ATCC 33591 (MIC 1 mg/L by our assay); clinical isolates exhibiting growth on screening media (MIC in parentheses, in mg/L, followed by MIC of subclone)—302 (1, 4), 354 (0.5, 1), 1150 (1,2), 1203 (1,2); clinical isolates with no growth on screening media (MIC in parentheses, in mg/L)—328 (1), 736 (1), 744 (1), 1149 (1). Isolates with growth on screening media are represented by solid lines; isolates with no growth are represented by broken lines. Isolates 354 and 1150 were cultured from the same patient, 14 months apart. b) Population analysis of an MRSA bloodstream isolate exhibiting heterogeneously reduced susceptibility to vancomycin, and its subclone, compared with reference strains. ATCC, MRSA reference strain ATCC 33591 (MIC 1 mg/L by our assay); PC3, VISA reference strain (published MIC 8 mg/L (*19*); MIC 4 mg/L by our assay); 1337, clinical isolate (MIC 1 mg/L); 1337d, subclone of 1337 grown on screening media (MIC 2 mg/L). While the population curve representing 1337d closely resembles that of PC3, this subclone did not meet MIC criteria for VISA. CFU, colony-forming units.

We performed population analysis on eight isolates (four that exhibited growth on screening media and four with no growth) to determine the utility or population analysis in distinguishing between these two types of isolates. The results, represented graphically in Figure 2a, do not enable such a distinction to be made. A shift in the curve is apparent, however, for two isolates with positive screening results from the same patient cultured 14 months apart. For the earlier isolate, the MIC of vancomycin was 0.5 mg/L. For the later isolate, cultured after several interval admissions for MRSA bacteremia in which the patient received vancomycin, the MIC was 1.0 mg/L, and an upwardly shifted population curve (signifying a greater proportion of the population with higher MICs of vancomycin) was observed. A subsequent population analysis, run on selected parent-subclone pairs for isolates that grew on screening media, demonstrated an upward shift in the population curve for the subclone versus the parent when a corresponding increase in MIC existed. A representative population curve is shown in Figure 2b.

### Epidemiologic Results

A comparison of patients with isolates with positive screening results (cases) to those with negative results (controls) demonstrated no significant differences in terms of age, sex distribution, coexisting chronic conditions, recent hospitalization, or hospital events before culture (Table 2). Similarly, no differences were found between the groups with respect to administration of any antibiotic, administration of vancomycin specifically, or number of days of vancomycin exposure before culture (Table 3). In the cohort analysis of the impact of the screening phenotype on patient outcomes, no differences were noted between case-patients and controls in deaths during hospitalization, discharge disposition, or time to discharge after culture for those who survived (Table 4).

**Table 2:** Descriptive characteristics of cohort

Characteristic	Cases, n=61 (%)	Controls, n=8 (%)	p	
Mean age (y)	61±17	64±14	0.21	
Male	37 (61)	53 (60)	1.00	
Diabetes mellitus	30 (49)	34 (39)	0.24	
Renal disease	20 (33)	29 (33)	1.00	
Hemodialysis	10 (16)	12 (14)	0.65	
Cardiovascular disease	41 (67)	56 (64)	0.73	
Pulmonary disease	19 (31)	35 (40)	0.30	
Hepatic disease	8 (13)	7 (8)	0.41	
HIV	3 (5)	3 (3)	0.69	
Prosthetic joint or valve, or permanent pacemaker	13 (21)	11 (13)	0.18	
Hospitalization at same institution <30 days preceding culture	51 (84)	66 (75)	0.23	
Surgery during admission before culture	17 (28)	20 (23)	0.56	
Intensive care unit stay during admission before culture	33 (54)	38 (43)	0.24	
Severity of illness score^a^			0.62	

^a^Severity of illness is based on modified McCabe criteria (23): 1, severe underlying coexisting chronic condition at imminent risk of death; 2, significant underlying coexisting chronic condition, not at imminent risk of death; 3, no significant underlying coexisting chronic condition.

**Table 3:** Antibiotic exposures during hospitalization before culture

Antibiotic exposure	Cases n=58 (%)	Controls n=84 (%)	p value

Administration of any antibiotic	27 (47)	45 (54)	0.50
Administration of vancomycin	10 (17)	14 (17)	1.00
Number of days receiving vancomycin (mean)	1.2±4.8	1.0±3.5	0.88

**Table 4:** Clinical outcomes




Outcome	Case-patients n=61 (%)	Controls n=87 (%)	p value (unadjusted)	p value (adjusted for severity of illness)

Discharged to home	13 (22)	22 (25)		
Discharged to an institution	32 (52)	42 (48)	0.83	0.67
Died during hospitalization	16 (26)	23 (26)		

Given the absence of standardized criteria for determining the screening phenotype, we further analyzed the data using a number of more stringent definitions. If we considered growth on screening media within 48 h with an associated increase in MIC among the subclones as the criteria for a positive screening result (n=46), again no significant predictors of the phenotype and no differences in outcomes were found. Twenty patients (13%) had isolates that grew on screening media within 24 h. Using this characteristic as the screening criterion, we found that the only significant predictor of this phenotype was intensive care unit stay before culture (OR 2.95; 95% confidence interval [CI] 1.07 to 8.15; p=0.05); no differences in outcomes were found (data not shown).

Finally, since the criteria for isolate collection were not identical in the two study institutions, we performed a subgroup analysis using data from Beth Israel Deaconess Medical Center patients alone (n=120). This analysis did not change the results of using the 48-h growth cutoff. Analysis of these data using the 24-h cutoff showed diabetes mellitus to be the only significant predictor of heterogeneously reduced susceptibility to vancomycin (OR 3.52; 95% confidence interval, 1.04 to 11.96; p=0.05), with no differences in outcomes (data not shown).

## Discussion

First reported in a clinical specimen from Japan in 1997 (*2*), VISA strains (MIC 8–16 mg/L) (*17*) have now been isolated in numerous countries around the world (*3*,*24*,*25*) and have been identified in several patients in the United States (*7*,*19*,*26*). These patients have in common extensive prior exposure to vancomycin, and their clinical courses are notable for a suboptimal response to this agent (*1*,*7*). Since VISA isolates are presumed to spread as do vancomycin-susceptible strains of *S. aureus*, their appearance has prompted the issuance of guidelines for their identification and control of transmission (*27*,*28*).

Shortly after the report of the first VISA isolate was published, Hiramatsu et al. reported an *S. aureus* isolate exhibiting heteroresistance to vancomycin (*6*). They found this phenotype of vancomycin heteroresistance to be widespread in Japanese university hospitals. In the ensuing 5 years, reports emerged of heteroresistant *S. aureus* in numerous countries (*6*–*16*).

Several areas of uncertainty have marked the subject of vancomycin heteroresistance since it was first described. First, no standardized definition exists, and investigators have defined it by using a variety of criteria (*6*,*8*,*11*,*16*,*29*,*30*). Second, the clinical significance of heteroresistance remains unclear. Although existing evidence supports the hypothesis that heteroresistant isolates have a greater likelihood of developing homogeneous intermediate resistance than do susceptible strains (*6*), and other data suggest an association between isolation of VISA and an adverse outcome (*7*), few studies have examined whether specific risk factors exist for infection with hetero-VISA, and whether such infection is associated with adverse outcomes. Those studies that have examined risk factors have had small sample sizes (*10*), inadequate generalizability (*8*), or a method of control selection that did not allow for direct comparison between patients with hetero-VISA and those infected with *S. aureus* isolates that were homogeneously susceptible to vancomycin (*13*). No study before ours has explored the clinical implications of varying degrees of vancomycin susceptibility in MRSA isolates that do not qualify as hetero-VISA.

This study addresses the prevalence of vancomycin heteroresistance among bloodstream MRSA isolates at two large, urban teaching hospitals and explores clinical correlates of bacteremia with isolates exhibiting heterogeneously reduced vancomycin susceptibility, as defined by growth on vancomycin screening agar (vancomycin 4 mg/L). We chose a screening method to encompass as broad an array of potentially heteroresistant isolates as possible. By subjecting subclones to agar dilution, however, we were deliberately conservative, in order to determine whether our isolates fulfilled Hiramatsu’s criteria for vancomycin heteroresistance in as unambiguous a manner as possible (*6*). Although none of our isolates were characterized as hetero-VISA under these strict criteria, over 40% had subpopulations capable of growth on our vancomycin-containing screening media. Such findings are plausible because *S. aureus* strains are known to differ in their propensity to develop reduced susceptibility to vancomycin (*31*). It is possible that some or all of the isolates from our cases are potential precursors of truly heteroresistant isolates (hetero-VISA), which may in turn be forerunners of VISA (*7*,*18*,*32*).

Howe et al. have criticized the method of screening for vancomycin heteroresistance in the presence of the antibiotic, followed by MIC testing of subclones, as being poorly reproducible and potentially selecting for, rather than simply detecting, resistant mutants (*29*). Regarding reproducibility, we repeated screening for 12 clinical isolates (6 with initially positive results, and 6 with negative results) at different times, using freshly made media each time. Ten (83%) of these had concordant results for the two tests (growth within 48 h on screening media with or without NaCl was the criterion for a positive result). Regarding the possibility of selection, even if this method does select for subclones that grow on screening media, its ability to do so may represent detection of a strain-specific phenomenon that could be of clinical importance if it occurs in vivo during vancomycin therapy.

Having identified the positive screening phenotype among our isolates, we sought to uncover clinical predictors of bacteremia with isolates exhibiting this phenotype, as well as to determine whether such bacteremia was associated with adverse outcomes. Our results were negative on both counts. The results were rendered more robust by remaining essentially unchanged even as we varied the definition of a positive screening result and performed an institution-based subgroup analysis. More stringent definitions of positivity other than those we explored (for example, a requirement of growth on non–salt-containing media), may have led us to undiscovered clinical differences between case-patients and controls.

These negative results can be interpreted in a number of ways. First, our study may have lacked statistical power to detect small differences between the groups in predictors and outcomes. Given the degree to which most of the p values deviate from statistical significance, however, analysis of a substantially larger cohort would be required to disprove such a claim. Moreover, our isolate cohort was highly clonal, as a single type accounted for 69% of isolates and the two most prominent types accounted for 84%. This degree of clonality among pathogenic isolates of MRSA may bias any attempted comparison of clinical features among the bacteremic patients (*33*).

Another possible explanation for our failure to detect outcome differences between case-patients and controls is the small degree of vancomycin exposure among all patients in the cohort before blood was drawn for culture. Only 17% (10 case-patients and 14 controls) of patients in the case and control groups received vancomycin before their blood was cultured, and the average amount of time that vancomycin was used by case-patients and controls before culture was approximately 1 day. Clinically, this finding is understandable: Most patients in the cohort had no reason to receive vancomycin before the growth of MRSA. Still, as pharmacologic data were not uniformly available before the patient’s hospitalization, we were able to focus on receipt of vancomycin during the index admission only, thereby perhaps limiting our ability to distinguish between study groups based on vancomycin exposure.

No difference may actually exist between bacteremia with VSSA strains that exhibit heterogeneously reduced susceptibility to vancomycin, as defined by growth on vancomycin, 4 mg/L screening agar, and homogeneously susceptible isolates. Such a conclusion would not rule out a clinical difference associated with hetero-VISA bacteremia; because our cohort contained no such cases, we cannot draw any conclusions regarding this question. Also, we may have not detected a difference in outcomes because we did not focus on the relevant ones. If, for example, heterogeneously reduced vancomycin susceptibility comes at the expense of a certain degree of virulence, as suggested by Burnie et al*.* on the basis of a mouse model (*34*), then we would not necessarily expect our case-patients to have more hospital deaths or even a greater length of stay in the hospital. We might, however, expect to find several years from now that case-patients will have a greater number of recurrent MRSA infections than will controls, or longer durations of bacteremia during such infections, due to failure of vancomycin to eradicate the organism effectively.

Our results show that despite a subtle phenotypic difference in the MRSA isolates of a large minority of the patients in our cohort, these patients were no different than the reference group with respect to clinical characteristics, antimicrobial use and other hospital exposures, and clinical outcomes. These results add weight to assertions that clinical microbiology laboratories need not routinely screen for vancomycin heteroresistance in *S. aureus* isolates with vancomycin MICs in the susceptible range (*2*,*8*). Additional studies with larger cohorts and a longer period of follow-up are needed to validate these findings, determine whether they apply to infection with true hetero-VISA, and evaluate outcomes suggestive of noneradicated, indolent infection.

## References

[1] Tenover FC, Biddle JW, Lancaster MV. Increasing resistance to vancomycin and other glycopeptides in *Staphylococcus aureus.* Emerg Infect Dis. 2001;7:327–32. 10.3201/eid0702.010237 11294734 PMC2631729

[2] Hiramatsu K, Hanaki H, Ino T, Yabuta K, Oguri T, Tenover FC. Methicillin-resistant *Staphylococcus aureus* clinical strain with reduced vancomycin susceptibility. J Antimicrob Chemother. 1997;40:135–6. 10.1093/jac/40.1.135 9249217

[3] Oliveira GA, Dell’Aquila AM, Masiero RL, Levy CE, Gomes MS, Cui L, Isolation in Brazil of nosocomial *Staphylococcus aureus* with reduced susceptibility to vancomycin. Infect Control Hosp Epidemiol. 2001;22:443–8. 10.1086/501932 11583214

[4] Centers for Disease Control and Prevention. *Staphylococcus aureus* resistant to vancomycin–United States, 2002. MMWR Morb Mortal Wkly Rep. 2002;51:565–7. 12139181

[5] Centers for Disease Control and Prevention. Public heath dispatch: vancomycin-resistant *Staphylococcus aureus*—Pennsylvania, 2002. MMWR Morb Mortal Wkly Rep. 2002;51:902. 12418544

[6] Hiramatsu K, Aritaka N, Hanaki H, Kawasaki S, Hosoda Y, Hori S, Dissemination in Japanese hospitals of strains of *Staphylococcus aureus* heterogeneously resistant to vancomycin. Lancet. 1997;350:1670–3. 10.1016/S0140-6736(97)07324-8 9400512

[7] Fridkin SK. Vancomycin-intermediate and –resistant *Staphylococcus aureus*: what the infectious disease specialist needs to know. Clin Infect Dis. 2001;32:108–15. 10.1086/317542 11118389

[8] Ariza J, Pujol M, Cabo J, Pena C, Fernandez N, Linares J, Vancomycin in surgical infections due to meticillin-resistant *Staphylococcus aureus* with heterogeneous resistance to vancomycin. Lancet. 1999;353:1587–8. 10.1016/S0140-6736(99)01017-X 10334262

[9] Geisel R, Schmitz FJ, Thomas L, Berns G, Zetsche O, Ulrich B, Emergence of heterogeneous intermediate vancomycin resistance in *Staphylococcus aureus* isolates in the Düsseldorf area. J Antimicrob Chemother. 1999;43:846–8. 10.1093/jac/43.6.846 10404328

[10] Marchese A, Balistreri G, Tonoli E, Debbia EA, Schito GC. Heterogeneous vancomycin resistance in methicillin-resistant *Staphylococcus aureus* strains isolated in a large Italian hospital. J Clin Microbiol. 2000;38:866–9. 10655401 10.1128/jcm.38.2.866-869.2000 PMC86227

[11] Chesneau O, Morvan A, El Solh N. Retrospective screening for heterogeneous vancomycin resistance in diverse *Staphylococcus aureus* clones disseminated in French hospitals. J Antimicrob Chemother. 2000;45:887–90. 10.1093/jac/45.6.887 10837445

[12] Bobin-Dubreux S, Reverdy ME, Nervi C, Rougier M, Bolmström A, Vandenesch F, Clinical isolate of vancomycin-heterointermediate *Staphylococcus aureus* susceptible to methicillin and in vitro selection of a vancomycin-resistant derivative. Antimicrob Agents Chemother. 2001;45:349–52. 10.1128/AAC.45.1.349-352.2001 11120996 PMC90291

[13] Wong SSY, Ho PL, Woo PCY, Yuen KY. Bacteria caused by staphylococci with inducible vancomycin heteroresistance. Clin Infect Dis. 1999;29:760–7. 10.1086/520429 10589883

[14] Howe RA, Bowker KE, Walsh TR, Feest TG, MacGowan AP. Vancomycin-resistant *Staphylococcus aureus.* Lancet. 1998;351:602. 10.1016/S0140-6736(05)78597-4 9492814

[15] Hubert SK, Mohammed JM, Fridkin SK, Gaynes RP, McGowan JE Jr, Tenover FC. Glycopeptide-intermediate *Staphylococcus aureus*: evaluation of a novel screening method and results of a survey of selected U.S. hospitals. J Clin Microbiol. 1999;37:3590–3. 10523558 10.1128/jcm.37.11.3590-3593.1999 PMC85700

[16] Tallent SM, Bischoff T, Climo M, Ostrowsky B, Wenzel RP, Edmond MB. Vancomycin susceptibility of oxacillin-resistant *Staphylococcus aureus* isolates causing nosocomial bloodstream infections. J Clin Microbiol. 2002;40:2249–50. 10.1128/JCM.40.6.2249-2250.2002 12037100 PMC130823

[17] National Committee for Clinical Laboratory Standards. Methods for dilution antimicrobial susceptibility tests for bacteria that grow aerobically, 5th edition: approved standard M7-A5. Villanova (PA): National Committee for Clinical Laboratory Standards, 2000.

[18] Sugino Y, Iinumab Y, Ichiyama S, Ito Y, Ohkawa S, Nakashima N, In vivo development of decreased susceptibility to vancomycin in clinical isolates of methicillin-resistant *Staphylococcus aureus.* Diagn Microbiol Infect Dis. 2000;38:159–67. 10.1016/S0732-8893(00)00186-3 11109014

[19] Sieradzki K, Roberts RB, Haber SW, Tomasz A. The development of vancomycin resistance in a patient with methicillin-resistant *Staphylococcus aureus* infection. N Engl J Med. 1999;340:517–23. 10.1056/NEJM199902183400704 10021472

[20] Lauderdale TL, Chapin KC, Murray PR. Reagents. In: Murray PR, Baron IJ, Pfaller MA, Tenover FC, Yolken RH, editors. Manual of clinical microbiology, 7th edition. Washington: American Society for Microibology; 1999. p. 1665–73.

[21] Tenover FC, Arbeit RD, Goering FV, Mickelsen PA, Murray BE, Persing DH, Interpreting chromosomal DNA restriction patterns produced by pulsed-field gel electrophoresis: criteria for bacterial strain typing. J Clin Microbiol. 1995;33:2233–9. 7494007 10.1128/jcm.33.9.2233-2239.1995 PMC228385

[22] Maslow J, Slutsky AM, Arbeit RD. The application of pulsed-field gel electrophoresis to molecular epidemiology. In: Persing DH, Smith TF, Tenover FC, White TJ, editors. Diagnostic molecular microbiology: principles and applications. Washington: American Society for Microbiology; 1993. p. 563–72.

[23] McCabeW. Jackson G. Gram-negative bacteremia. Arch Intern Med. 1962;110:847–55.

[24] Ploy MC, Grélaud C, Martin C, de Lumley L, Denis F. First clinical isolate of vancomycin-intermediate *Staphylococcus aureus* in a French hospital. Lancet. 1998;351:1212. 10.1016/S0140-6736(05)79166-2 9643727

[25] Bierbaum G, Fuchs K, Lenz W, Szekat C, Sahl HG. Presence of *Staphylococcus aureus* with reduced susceptibility to vancomycin in Germany. Eur J Clin Microbiol Infect Dis. 1999;18:691–6. 10.1007/s100960050380 10584894

[26] Smith TL, Pearson ML, Wilcox KR, Cruz C, Lancaster MV, Robinson-Dunn B, Emergence of vancomycin resistance in *Staphylococcus aureus.* N Engl J Med. 1999;340:493–501. 10.1056/NEJM199902183400701 10021469

[27] Centers for Disease Control and Prevention. Interim guidelines for prevention and control of staphylococcal infection associated with reduced susceptibility to vancomycin. MMWR Morbid Mortal Wkly Rep 1997;46:626–8,635–6. 9218649

[28] Chadwick PR, Wooster SL. Glycopeptide resistance in *Staphylococcus aureus.* J Infect. 2000;40:211–7. 10.1053/jinf.2000.0651 10908014

[29] Howe RA, Wootton M, Walsh TR, Bennett PM, MacGowan AP. Heterogeneous resistance to vancomycin in *Staphylococcus aureus.* J Antimicrob Chemother. 2000;45:130–1. 10.1093/jac/45.1.130 10629026

[30] Hanaki H, Inaba Y, Sasaki K, Hiramatsu K. [A novel method of detecting *Staphylococcus aureus* heterogeneously resistant to vancomycin (hetero-VRSA)] (article in Japanese). Jpn J Antibiot. 1998;51:521–30. 9836124

[31] Pfeltz RF, Singh VK, Schmidt JL, Batten MA, Baranyk CS, Nadakavukaren MJ, Characterization of passage-selected vancomycin-resistant *Staphylococcus aureus* strains of diverse parental backgrounds. Antimicrob Agents Chemother. 2000;44:294–303. 10.1128/AAC.44.2.294-303.2000 10639353 PMC89674

[32] Hiramatsu K. Vancomycin-resistant *Staphylococcus aureus*: a new model of antibiotic resistance. Lancet Infect Dis. 2001;1:147–55. 10.1016/S1473-3099(01)00091-3 11871491

[33] Cimolai N. Are all MRSA made equal? Can J Microbiol. 2002;48:560–6. 10.1139/w02-045 12166684

[34] Burnie J, Matthews R, Jiman-Fatami A, Gottardello P, Hodgetts S, D’arcy S. Analysis of 42 cases of septicemia caused by an epidemic strain of methicillin-resistant *Staphylococcus aureus:* evidence of resistance to vancomycin. Clin Infect Dis. 2000;31:684–9. 10.1086/314035 11017816

