# Correction Vol. 9, No. 6

**DOI:** 10.3201/eid1001.C11001

**Published:** 2004-01

**Keywords:** errata, erratum, correction

In "Clinical Implications of Varying Degrees of Vancomycin Susceptibility in Methicillin-Resistant Staphylococcus aureus Bactermia, by Mitchell J. Schwaber et al., errors occurred in some reference numbers. In the Discussion, fourth paragraph, p. 661, the last sentence should read as follows: 'It is possible that some or all of the isolates from our cases are potential precursors of truly heteroresistant isolates (hetero-VISA), which may in turn be forerunners of VISA (6,18,32)." In the final paragraph, pp. 662-663, the next to last sentence should read as follows:

"These results add weight to assertions that clinical microbiology laboratories need not routinely screen for vancomycin heteroresistance in S. aureus isolates with vancomycin MICs in the susceptible range (1,7).”

The corrected article can be found online at http://wwwnlc.cdc.gov/eid/article/9/6/03-0001_article.htm. 

